# A Recombinant Thermophilic and Glucose-Tolerant GH1 β-Glucosidase Derived from Hehua Hot Spring

**DOI:** 10.3390/molecules29051017

**Published:** 2024-02-26

**Authors:** Qian Zhu, Yuying Huang, Zhengfeng Yang, Xingci Wu, Qianru Zhu, Hongzhao Zheng, Dan Zhu, Zhihua Lv, Yirui Yin

**Affiliations:** 1 College of Agriculture and Biological Science, Dali University, Dali 671003, China; 13688714085@163.com (Q.Z.); 13471902743@163.com (Y.H.); yzfwym110803@163.com (Z.Y.); 18508819172@163.com (X.W.); 19533007216@163.com (Q.Z.); 19187653632@163.com (H.Z.); dzhudali@163.com (D.Z.); 2 Key Laboratory of Bioinformatics and Computational Biology, Department of Education of Yunnan Province, Dali University, Dali 671003, China; 3 Cangshan Forest Ecosystem Observation and Research Station of Yunnan Province, Dali University, Dali 671003, China

**Keywords:** hot spring, metagenomic, β-glucosidase, thermophilic, glucose tolerance

## Abstract

As a crucial enzyme for cellulose degradation, β-glucosidase finds extensive applications in food, feed, and bioethanol production; however, its potential is often limited by inadequate thermal stability and glucose tolerance. In this study, a functional gene (*lq-bg5*) for a GH1 family β-glucosidase was obtained from the metagenomic DNA of a hot spring sediment sample and heterologously expressed in *E. coli* and the recombinant enzyme was purified and characterized. The optimal temperature and pH of LQ-BG5 were 55 °C and 4.6, respectively. The relative residual activity of LQ-BG5 exceeded 90% at 55 °C for 9 h and 60 °C for 6 h and remained above 100% after incubation at pH 5.0–10.0 for 12 h. More importantly, LQ-BG5 demonstrated exceptional glucose tolerance with more than 40% activity remaining even at high glucose concentrations of 3000 mM. Thus, LQ-BG5 represents a thermophilic β-glucosidase exhibiting excellent thermal stability and remarkable glucose tolerance, making it highly promising for lignocellulose development and utilization.

## 1. Introduction

Cellulose stands as the most abundant renewable natural polysaccharide in plant biomass [1], serving as an essential raw material across various industries including paper, textile, and food. Achieving complete degradation of cellulose into glucose necessitates the synergistic catalysis of exoglucanase, endoglucanase, and β-glucosidase [2]. Endoglucanase operates by hydrolyzing cellulose chains internally, whereas exoglucanase degrades cellulose from either the reducing or non-reducing end, yielding cellulose oligosaccharides. These oligosaccharides are subsequently hydrolyzed by β-glucosidase to yield glucose [3]. Serving as the rate-limiting enzyme in cellulose degradation, β-glucosidase mitigates feedback inhibition exerted by cellulose oligosaccharides on both endoglucanase and exoglucanase [4], a pivotal aspect ensuring the efficient utilization of cellulose. β-Glucosidase finds numerous critical applications in food and beverage production, biofuels, and pharmaceuticals [5,6,7,8,9]. However, the activity of β-glucosidase is susceptible to factors such as temperature, pH, inhibitors, or glucose concentration, thereby restricting its utility across various sectors including food and industry.

Currently, industrially applied β-glucosidases are predominantly sourced from fungi, such as *Trichoderma reesei*, *Phanerochaete chrysosporium*, and others [10,11,12]. However, most fungal-derived β-glucosidases exhibit rapid inactivation at temperatures exceeding 50 °C [13,14], limiting their use in high-temperature conditions. Since the end product of cellulose hydrolysis by β-glucosidases is glucose, and most β-glucosidases are sensitive to high glucose concentrations with Ki values ranging from 0.5 to 100 mM [15], they are prone to inhibition by glucose, resulting in the accumulation of cellobiose and oligosaccharides [16]. β-Glucosidases also exhibit limited survival in highly acidic or alkaline conditions, with the majority having an optimal pH range between 4.0 and 7.5 and remaining stable within pH 4.0–9.0 [17].

In industrial applications, thermophilic archaea and bacteria are particularly sought after due to their ability to thrive at temperatures exceeding 70 °C, and their enzymes exhibit thermophilic properties [18,19]. Notably, the β-glucosidase from the submarine *Thermotoga maritima* can function optimally at temperatures as high as 90 °C [20]. Enzymes adapted to high temperatures offer advantages in reducing the risk of contamination during application, and microorganisms capable of surviving extreme temperatures and environments serve as crucial sources of enzymes for heterogeneous biomass conversion processes. While engineering similar properties in mesophilic enzymes often requires complex engineering and optimization, enzymes from extremophilic organisms frequently possess such properties naturally [21,22]. When looking for enzymes that can hydrolyze biomass at high temperatures, enzymes derived from these extreme microorganisms are usually of better properties than those from ambient sources. For instance, novel β-glucosidase from the hot spring metagenome demonstrates increased thermal stability and tolerance to glucose and ethanol [23]. Some β-glucosidases exhibit glucose tolerance, such as those from *Aspergillus tubingensis* CBS 643.92, *A. oryzae*, *A. niger* CCRC 31494, *A. foetidus*, and marine microbial metagenome, which have shown high Ki values of 600 mM, 1390 mM, 543 mM, 520 mM, and 1000 mM, respectively [24]. However, despite their glucose tolerance, these β-glucosidases demonstrate low hydrolytic activity towards cellobiose. Therefore, having a β-glucosidase with both high glucose tolerance and high hydrolytic activity towards cellobiose is advantageous for alleviating the feedback inhibition of the products during hydrolysis and promoting the efficient degradation of cellulosic biomass. Β-Glucosidase exhibits several shortcomings, including low activity, glucose intolerance, and poor stability. These limitations render β-glucosidase inadequate for meeting the demands of practical production. Therefore, it is crucial to identify β-glucosidases with high stability, thermophilic properties, acidophilic characteristics, and resistance to inhibition by glucose.

In this study, a GH1 β-glucosidase gene (*lq-bg5*) was obtained from the metagenomic DNA data of Tengchong Hehua spring sediment. Subsequently, it was cloned into *E. coli* DH5α for heterologous expression and protein purification. Enzymatic characterization of LQ-BG5 revealed its thermophilic, acidophilic, and glucose-tolerant properties. These findings suggest its promising potential in industrial applications and the promotion of lignocellulose degradation.

## 2. Results

### 2.1. Sequence Analysis, Heterologous Expression, Protein Isolation, and Purification

**Figure 1:**
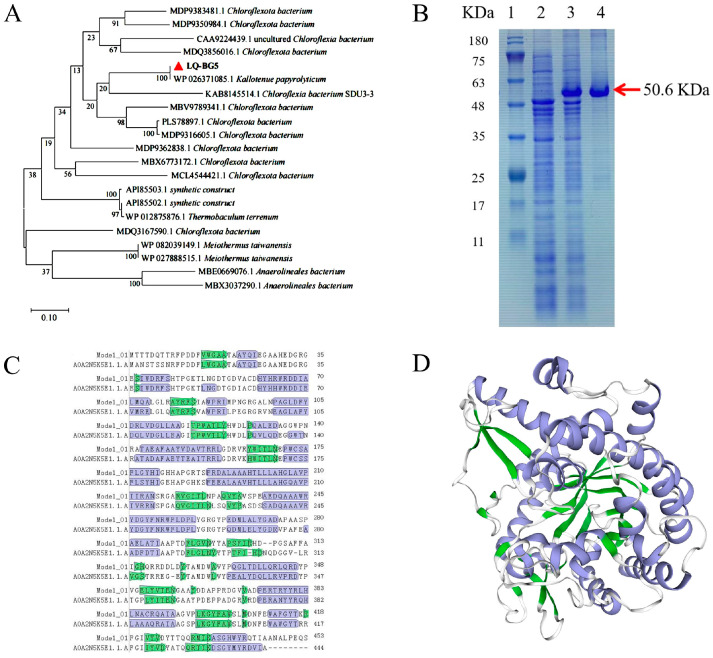
(**A**) Phylogenetic dendrogram obtained by maximum likelihood analysis based on amino acid sequences showing the phylogenetic position of LQ-BG5 with related β-glucosidases. Bootstrap values (expressed as a percentage of 1000 replications) are given at nodes. (**B**) SDS-PAGE analysis of LQ-BG5. Lane 1, protein molecular weight marker; Lane 2, protein of *E. coli* DH5α prior to induction; Lane 3, total protein of *E. coli* DH5α/pSHY211- LQ-BG5; Lane 4, purified LQ-BG5 protein. Complete SDS-PAGE image was shown in Supplementary Figure S4. (**C**) Comparison of the amino acid sequences of LQ-BG5 with the homology model. (**D**) LQ-BG5 homology model. Purple and green structures represent α-helix and β-fold, respectively.

The sequencing of DNA extracted from the Hehua hot spring’s sediment (with temperature of 65.2 °C and a pH of 8.2) resulted in a comprehensive data set that included 3.2 Gbp and featured 26,860 contigs of >500 bp in length. From these data, it was possible to identify a total of 123,938 distinct genes. A GH1 family functional β-glucosidase gene of size 1362 bp was obtained in the metagenomic DNA data, named *lq-bg5*. The gene fragment was amplified by PCR using the metagenomic DNA of the hot spring as a template. Under UV light, the PCR products exhibited clear and bright bands (Figure S1). Subsequently, accurate recovery was achieved through gel extraction. The *lq-bg5* gene ligated to pSHY211 was successfully transformed into *E. coli* DH5α (Figure S2). The *E. coli* clone containing the recombinant plasmid pSHY211-LQ-BG5 was screened by colony PCR and Sanger sequencing. The sequencing result of the *lq-bg5* gene was consistent with the metagenomic sequencing data. The complete nucleotide sequence of *lq-bg5* was submitted to GenBank (NCBI) under accession number OR724869. The phylogenetic tree was constructed by comparing the protein sequences of LQ-BG5 using NCBI BLASTp. It was found that the highest sequence identity (100%) was observed with GH1 family β-glucosidase originating from *Kallotenue papyrolyticum* (WP 026371085.1). Additionally, there was a sequence identity of 72.13% with β-glucosidase originating from *Chloroflexota bacterium* (PLS78897.1), and a sequence identity of 71.46% with β-glucosidase from *Chloroflexota* origin (MBV9789341.1). The phylogenetic tree analysis also revealed that LQ-BG5 was grouped together with β-glucosidases derived from *Kallotenue papyrolyticum* (WP 026371085.1) and *Chloroflexota bacterium* (KAB8145514.1) in a specific branch (Figure 1A).

The protein sequence of LQ-BG5 was analyzed using the SWISS-MODEL online software (https://swissmodel.expasy.org/, accessed on 10 November 2023). The model demonstrating the highest amino acid sequence identity (73.30%) and global model quality estimation (GMQE) score of 0.95 was selected as the final template for comparing the amino acid sequences (Figure 1C). The homology model of LQ-BG5 clearly illustrates the characteristic triosephosphate isomerase (TIM) barrel-like structure, comprising a peptide backbone with eight outer α helixes and eight inner parallel β chains (Figure 1D). The three-dimensional model of β-glucosidases LQ-BG5 shows that it is very similar to other GH1 β-glucosidases with known structures, all having the same (β/α)_8_ structure (Figure S3). Consequently, the recombinant β-glucosidase LQ-BG5 is classified within the GH1 family.

Sequence analysis revealed that LQ-BG5 harbored the catalytic module of glycoside hydrolase family 1 (GH1) without a signal peptide, possessing a theoretical isoelectric point (pI) of 5.59 and a theoretical molecular weight of 50.6 kDa. The *lq-bg5* gene was cloned and heterologously expressed in *Escherichia coli* DH5α. The β-glucosidase LQ-BG5 protein demonstrated conformity with the theoretical prediction (50.6 kDa) following purification on a Ni-NTA column and analysis of the protein on SDS-PAGE, confirming the successful purification of the protein (Figure 1B).

### 2.2. Enzyme Properties

#### 2.2.1. Optimum Temperature and Thermal Stability of Recombinant β-Glucosidase

**Figure 2:**
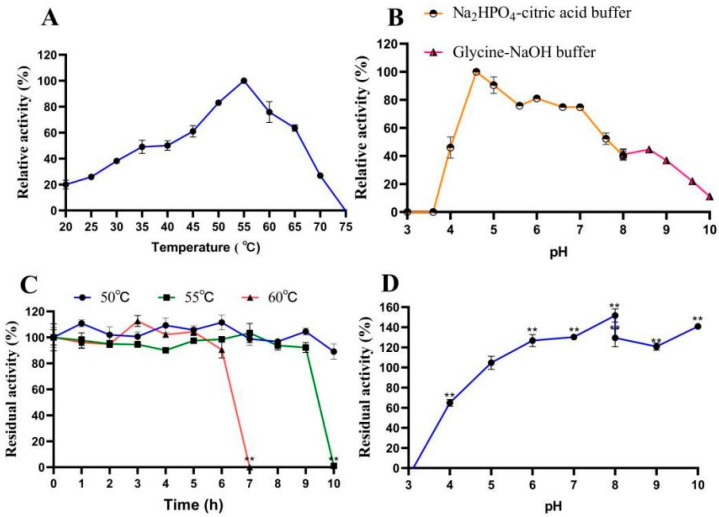
Effects of temperature and pH on the activity and stability of the recombinant LQ-BG5. (**A**) Temperature effect on the activity of LQ-BG5. (**B**) pH effect on the activity of LQ-BG5. (**C**) The effect of temperature on stability at different temperatures (50, 55, and 60 °C) for 0, 1, 2, 3, 4, 5, 6, 7, 8, 9, and 10 h. (**D**) The effect of pH on stability. Values represent the mean of three biological replicates. Error bars represent the mean ± SEM of three biological replicates. Significance analysis was performed for the effect of temperature and pH on enzyme stability. ** *p* < 0.01. The primary activity was taken as 100%. In total, 100% = 7.0 ± 2.1 U/mg.

**Figure 3:**
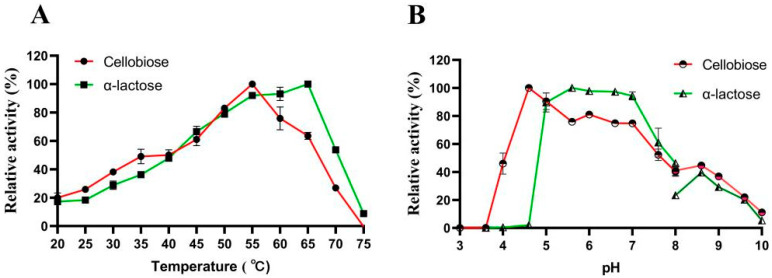
Effect of temperature and pH on the activity of recombinant LQ-BG5 when cellobiose and α-lactose were used as substrates. (**A**) Temperature effect on the activity of LQ-BG5. (**B**) pH effect on the activity of LQ-BG5. Error bars represent the mean ± SEM of three biological replicates.

When cellobiose served as the substrate, the recombinant β-glucosidase LQ-BG5 displayed its highest activity at 55 °C and maintained over 60% relative activity within the range of 45 °C to 65 °C (Figure 2A). Notably, LQ-BG5 retained more than 90% activity after 9 h of incubation at 50 °C and 55 °C, and after 6 h of incubation at 60 °C. The half-life of LQ-BG5 at the optimal temperature of 55 °C is 9.5 h. LQ-BG5 demonstrated significant thermal stability. These findings suggest that the enzyme exhibits characteristics of a thermophilic and heat-resistant β-glucosidase (Figure 2C). Furthermore, when α-lactose was utilized as the substrate, the optimal temperature for LQ-BG5 activity was determined to be 65 °C, with the relative activity also remaining above 60% within the range of 45 °C to 65 °C. Interestingly, LQ-BG5 displayed a similar trend of enzyme activity within the temperature range of 20 °C to 75 °C, whether cellobiose or α-lactose was used as the substrate (Figure 3A).

#### 2.2.2. Optimum pH and pH Stability of Recombinant β-Glucosidase

When cellobiose served as the substrate, the optimum pH for LQ-BG5 activity was determined to be 4.6, with the enzyme retaining approximately 80% relative activity within the pH range of 4.6 to 7.0, indicating a broad pH adaptability (Figure 2B). Following a 12 h incubation in buffers ranging from pH 3.0 to 10.0, the enzyme activity increased with the rising pH levels, which was particularly evident in buffers with pH values ranging from 5.0 to 10.0, where the enzyme activity was significantly enhanced. Specifically, in Na_2_HPO_4_-citric acid at pH 8.0, the enzyme activity was activated 1.52 times (Figure 2D). Moreover, when α-lactose was utilized as the substrate, the optimal pH for the recombinant β-glucosidase LQ-BG5 activity was found to be 5.6. The enzyme was rendered inactive at pH 4.6 (Figure 3B), while maintaining a relatively stable state of more than 80% relative activity within the pH range of 5.0 to 7.0.

#### 2.2.3. Effect of Metal Ions and Chemical Reagents on Enzyme Stability

As illustrated in Table 1, at an ion concentration of 1 mM and a chemical reagent concentration of 0.1%, the enzyme activities were slightly inhibited or not inhibited, except for SDS, which had a strong inhibitory effect on the enzyme activities, maintaining more than 85% of the relative activity. At higher concentrations of ions (10 mM) and chemicals (1%), several ions and chemicals strongly inhibited the enzyme activity, with complete inhibition observed in the presence of 10 mM Co^2+^, Ag^+^, and 1% SDS. Notably, the enzyme activity remained above 90% in the presence of 10 mM and 1 mM of K^+^, Ca^2+^, Zn^2+^, Cu^2+^, or 0.1% and 1% EDTA, indicating tolerance of LQ-BG5 to these ions or chemical reagents.

#### 2.2.4. Effect of Glucose Concentration on Enzyme Activity

**Figure 4:**
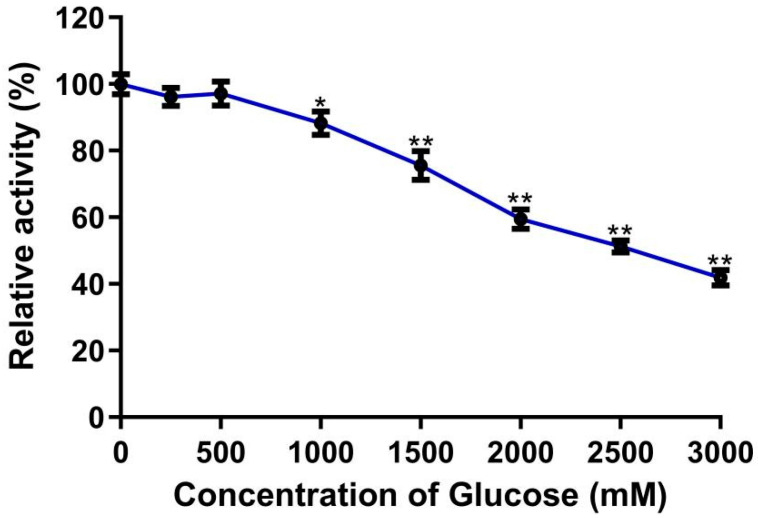
Effect of glucose concentration on the activity of recombinant LQ-BG5. Values represent the mean of three biological replicates. Error bars represent the mean ± SEM of three biological replicates. ** *p* < 0.01, * *p* < 0.05. The primary activity was taken as 100%. In total, 100% = 7.0 ± 2.1 U/mg.

The glucose tolerance of LQ-BG5 was assessed using 1 mM pNPG as the substrate. The observed trend indicated a gradual decrease in the relative enzyme activity with the increasing glucose concentration yet remaining at 75% when the glucose concentration reached 1500 mM. Notably, the relative enzyme activity remained above 40% even up to a glucose concentration of 3000 mM (Figure 4). The Ki value of the recombinant β-glucosidase LQ-BG5 was determined to be 2114.5 mM. These findings underscore LQ-BG5 as a glucose-tolerant β-glucosidase.

#### 2.2.5. Substrate Specificity and Enzyme Kinetic Parameters of LQ-BG5

The substrate specificity analysis of LQ-BG5 revealed its highest hydrolytic activity towards pNPG (24.1 ± 10.8 U/mg), with some degradation activity observed towards α-lactose (13.7 ± 1.5 U/mg) and cellobiose (7.0 ± 2.1 U/mg). However, no hydrolytic activity was detected towards the remaining substrates, including sucrose, corn cob xylan, avicel, wheat bran xylan, CMC, bagasse xylan, and beechwood xylan (Table 2).

**Figure 5:**
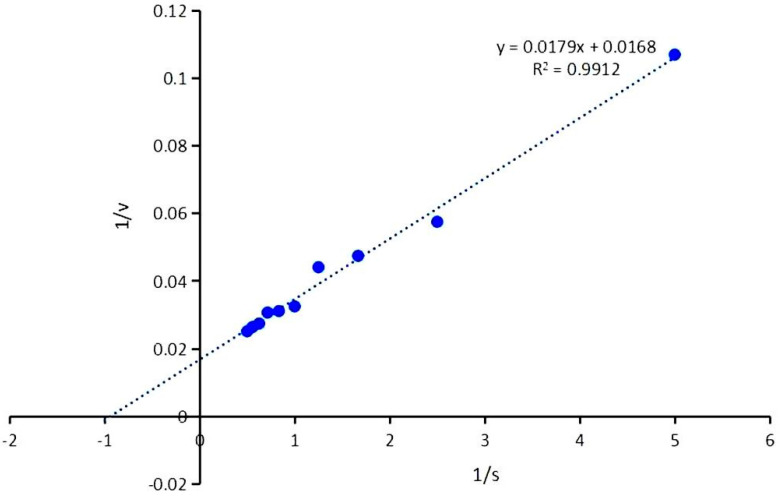
Lineweaver–Burk double-inverse plot of LQ-BG5 with cellobiose as substrate.

**Figure 6:**
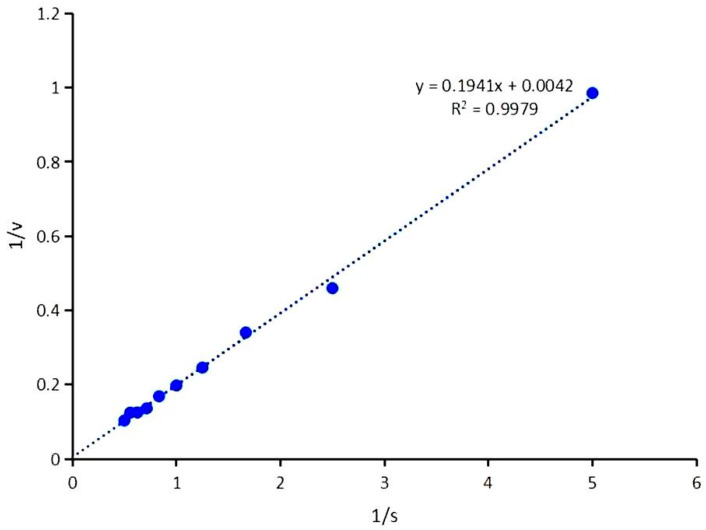
Lineweaver–Burk double-inverse plot of LQ-BG5 with α-lactose as substrate.

The kinetic parameters of the recombinant β-glucosidase were determined using the Michaelis–Menten equation. For the substrate cellobiose, the Km and Vmax values of the recombinant β-glucosidase LQ-BG5 were calculated as 3.11 mM and 59.52 μmol/min/mg, respectively, under optimal temperature and pH conditions (Figure 5). When α-lactose was employed as the substrate, the kinetic parameters Km and Vmax were found to be 128.27 mM and 238.10 μmol/min/mg, respectively (Figure 6).

## 3. Discussion

Studies have demonstrated that β-glucosidase derived from bacteria is predominantly found within the GH1 and GH3 families [25]. The protein sequence of LQ-BG5 shares the highest protein sequence identity (100%) with the GH1 β-glucosidase originating from *Kallotenue papyrolyticum* (WP 026371085.1). *Kallotenue papyrolyticum* is a newly discovered bacterial strain isolated from the Great Boiling Spring, known for its cellulose decomposition capabilities. This strain exhibits a growth temperature range of 45–65 °C, with an optimum temperature of 55 °C, and a growth pH range of 5.6–9.0, with an optimal pH of 7.5 [26]. Interestingly, these conditions are akin to the optimal temperature and pH (55 °C, pH 4.6) observed for β-glucosidase LQ-BG5 derived from the Hehua hot spring environment in Tengchong, China. This similarity further suggests that microbial distribution is evolutionarily global rather than regionally unique.

The optimum reaction temperature of LQ-BG5 in this study is 55 °C, and after incubation at 50 °C, 55 °C, and 60 °C for 10 h, the activity of about 100% is still maintained at 50 °C, and the activity was inactivated after 7 h at 60 °C, indicating that it is a thermophilic enzyme with good thermal stability. These findings suggest that LQ-BG5 is a thermophilic enzyme with notable thermal stability. Thermally stable β-glucosidase enzymes are crucial for various biotechnological applications, including those in the food and beverage industry and industrial processes utilizing lignocellulose [19]. The robust thermal stability of β-glucosidase not only facilitates cellulose hydrolysis but also enhances the reactant solubility, accelerates catalytic reaction rates, and reduces contamination risks, making it highly desirable in biomass processing [27]. Although LQ-BG5 shares a similar optimum temperature with most thermophilic β-glucosidases, it exhibits superior thermal stability compared to some reported counterparts. For instance, β-glucosidase from *Scytalidium thermophilum* exhibits a drastic decrease to less than 20% of enzyme activity after 1 h of incubation at 55 °C [28], while β-glucosidase from *Meyerozyma guilliermondii* shows a decrease to 90% of enzyme activity after 80 min of incubation at 55 °C [29]. These comparisons highlight the potential exploitation value of LQ-BG5 in cellulose industrial production.

β-Glucosidase possessing both thermophilic and acidophilic properties holds significant value in industrial applications [30]. Typically, β-glucosidases exhibit an active pH range between 4.0 and 7.5, and they tend to maintain stability within the pH range of 4.0 to 9.0 [17]. In this study, the optimum reaction pH of LQ-BG5 was determined to be 4.6, with the enzyme demonstrating approximately 80% relative activity within the pH range of 4.6 to 7.0. Following incubation in buffer solutions ranging from pH 5.0 to 10.0 for 12 h, an increase in the relative activity was observed and the pH tolerance and stability were good. Interestingly, the optimal pH was similar to the results of the β-glucosidase Bgl_M_ from a hot spring metagenomic (pH = 5) [23]. Furthermore, LQ-BG5 exhibited greater tolerance after 12 h of treatment at each pH compared to β-glucosidase (Bgl1973) from *Leifsonia* sp. ZF2019, whose enzyme activity dropped below 60% at pH 8 after 12 h of incubation [31]. The fact that β-glucosidase can tolerate a low pH and remain relatively stable is highly advantageous in some lignocellulose degradation applications, which are often acidic [32]. Consequently, LQ-BG5 shows promising potential for cellulose processing in acidic environments.

β-Glucosidases play a crucial role in various industrial applications. However, their catalytic activity is often hindered by the inhibitory effect of glucose, the hydrolysis product. Consequently, the reduced catalytic efficiency of β-glucosidases limits their industrial utility. To address this challenge, the selection of glucose-tolerant β-glucosidases capable of maintaining enzyme activity at high glucose concentrations has been prioritized, facilitating cellulose bioprocessing. Notably, β-glucosidases belonging to the GH1 family exhibit a higher glucose tolerance compared to those of GH3 [33]. The recombinant β-glucosidase LQ-BG5, a member of the GH1 family, demonstrates significant glucose tolerance. LQ-BG5 maintains activity above 50% even at an added glucose concentration of 2500 mM, with a determined Ki value of 2114.5 mM. According to the classification proposed by Cao et al. [16] and expanded by Salgado et al. [34], LQ-BG5 can be categorized as a type II enzyme capable of tolerating glucose. In general, most β-glucosidases from microbial sources lose most of their activity at glucose concentrations above 200 mM [24]. For instance, *Penicillium brasiliensis* produces a glucosidase rBGLPb with a Ki value of 2.3 mM, and *Aspergillus* sp. N188BG with a Ki value of 3.12 mM [35,36]. However, LQ-BG5 maintains approximately 41% activity even at a glucose concentration of 3000 mM, demonstrating remarkable glucose tolerance and strong potential for applications in food processing and industrial production.

The Km of LQ-BG5 was 3.11 mM when cellobiose was used as the substrate, which is stronger than most reported β-glucosidase substrate affinities using cellobiose as the substrate. For instance, β-glucosidase from *Thermoanaerobacterium aotearoense* exhibits a Km value of 25.45 mM [37], while β-glucosidase from the hyperthermophilic archaeon *Thermococcus* sp. has a Km value of 16.48 mM [38]. Moreover, recombinant β-glucosidase LQ-BG5 is active against α-lactose. This multifunctional characteristic suggests that LQ-BG5 possesses a broader range of industrial applications. The results of the present study were similar to those of MtBgl3c, a β-glucosidase of *Myceliophthora thermophila* origin, in that both were active against pNPG, cellobiose, and lactose [39]. Additionally, β-glucosidase Bgl_M_ from a hot spring metagenomic displays considerable hydrolysis potential for cellobiose and lactose, along with activity against various synthetic substrates [23]. In summary, the properties elucidated for LQ-BG5 in this study position it as a favorable candidate among reported β-glucosidases (Table 3), boasting several desirable traits suitable for industrial applications [1,23,24,29,31,38,40,41,42].

## 4. Materials and Methods

### 4.1. Culture Medium, Strain, and Plasmid

Luria broth (LB) medium: yeast extract 5 g/L, tryptone 10 g/L, NaCl 10 g/L, kanamycin was used as an antibiotic, and its final concentration was 100 μg/mL. The LB solid medium was supplemented with 2% agar.

The *E. coli* DH5α strain (obtained from Shenzhen KT Life Technology Co., Ltd., Shenzhen, China) and the constitutive expression plasmid pSHY211 vector (maintained in the laboratory) were employed for gene cloning and expression. *Eco*R I and *Hin*d III enzymes for double digestion were procured from Thermo Fisher Scientific, New York, NY, USA. The pEASY-Uni Seamless Cloning and Assembly Kit was obtained from TransGen Biotech, Beijing, China.

### 4.2. Sample Collection, Metagenomic Sequencing, and Sequence Analysis

The sediment samples (65.2 °C, pH 8.2) were collected from the Hehua hot spring (24.908284° N, 98.389563° E), of Tengchong City, Yunnan Province, then frozen in dry ice. The metagenomic DNA was extracted with the Power Soil Kit (MOBIO DNeasy PowerSoil Kit, New York, NY, USA) according to the manufacturer’s instructions. The metagenomic sequencing of the Hehua hot spring was conducted using the HiSeq 2500 instrument at GENWIZ in Suzhou. The sequences were investigated using the IMG server (https://img.jgi.doe.gov/cgi-bin/mer/main.cgi, accessed on 1 February 2023). To analyze the functions of individual genes and ORFs, the COG, KEGG, and Pfam databases were employed. We obtained a total of 11 β-glucosidase gene sequences belonging to the GH1 family. The genes were amplified by PCR using metagenomic DNA as a template, cloned, sequenced, aligned with the metagenomic sequence, and eventually subjected to heterologous expression and enzymatic property characterization. Among these, four genes were successfully heterologously expressed and validated through sequencing alignment. Initial assessment of the expression activity of these four genes allowed us to select one with higher activity for subsequent enzymatic property studies. The sequence of this β-glucosidase gene (named *lg-bg5*) was obtained from the metagenomic DNA data. The metagenomic data are deposited in the NCBI RefSeq repository with the accession number PRJNA1015443. 

A glycoside hydrolase GH1 β-glucosidase functional gene (*lg-bg5*) was obtained from the metagenomic DNA data, and its DNA and protein sequences were analyzed using BLASTx and BLASTp online software (http://blast.ncbi.nlm.nih.gov/Blast.cgi, accessed on 10 November 2023). Additionally, SignalP (https://services.healthtech.dtu.dk/services/SignalP-5.0/, accessed on 10 November 2023) was used for the prediction of signal peptides. Primary structures of amino acid sequences were deduced and analyzed using EXPASY tools (https://web.expasy.org/translate/, accessed on 10 November 2023). The protein sequence of LQ-BG5 was compared in NCBI BLASTp, and the phylogenetic tree was constructed using the maximum likelihood method (ML) and Poisson correction model in MEGA 7 software [43]. The protein sequence was analyzed using SWISS-MODEL (https://swissmodel.expasy.org, accessed on 10 November 2023) online software, and protein sequences with the highest sequence identity were used for modeling.

### 4.3. Gene Amplification, Cloning, and Recombinant Vector Construction

Using the metagenomic DNA as a template, PCR amplification of the *lq-bg5* gene was conducted with the following primers: *lq-bg5*-F (CATCATCATCATCATCATGAAATGACCACCACCGACCAGACT), *lq-bg5*-R (GTGCTCGAGTGCGGCCGCAAGTCAGCTCTCTGTTCCGGCAGAGC). The PCR program consisted of denaturation at 94 °C for 3 min, followed by 30 cycles at 98 °C for 15 s, 58 °C for 30 s, and 72 °C for 60 s, and then a final incubation at 72 °C for 5 min for the final extension. The underlined sequence represents a homologous recombinant fragment of the pSHY211 vector [44] that was previously digested with *Eco*R I and *Hin*d III. The PCR product was identified by 1.0% agarose gel electrophoresis. Gel recovery of bright bands was performed using the SanPrep Column DNA Gel Extraction Kit (Sangon Biotech (Shanghai) Co., Ltd., Shanghai, China). 

The purified PCR product was ligated to the pSHY211 vector, which had been previously digested by *Eco*R I and *Hin*d III, using the pEASY-Uni Seamless Cloning and Assembly Kit (TransGen Biotech, Beijing, China) to construct the recombinant plasmid pSHY211-LQ-BG5. DNA linking products were introduced into *E. coli* DH5α receptor cells by Ca^2+^ chemical transformation method. Positive clones without green fluorescence were screened with kanamycin (100 µg/mL) LB solid medium. The positive clones were screened by colony PCR and Sanger sequencing, to obtain the recombinant plasmid pSHY211-LQ-BG5. The sequencing results were compared with the metagenomic DNA data.

### 4.4. Heterologous Expression and Purification of LQ-BG5

The recombinant *E. coli* DH5α with pSHY211-LQ-BG5 was cultured into 200 mL LB medium containing 100 µg/mL kanamycin. The culture was then incubated at 37 °C with shaking at 180 rpm for 8 h to promote *E. coli* growth. Subsequently, the culture was transferred to 25 °C and shaken at 180 rpm for 12 h to allow for sufficient cellular protein production. *E. coli* cells were harvested by centrifugation at 12,000× *g*, 4 °C for 20 min [44]. Following centrifugation, the collected *E. coli* cells were initially resuspended in a centrifuge tube containing PBS solution supplemented with 10 mM imidazole (pH 7.6). Subsequently, the cells were sonicated in an ice–water mixture for 30 min and centrifuged at low temperature (4 °C) at 12,000× *g* for 20 min to obtain the supernatant, which served as the crude enzyme. The crude enzyme was then subjected to purification using a Ni-NTA column following the method previously reported by Huang et al. [45]. The protein concentration was estimated using Bradford reagent (Sangon Biotech (Shanghai) Co., Ltd., Shanghai, China) [46], with bovine serum albumin serving as the standard. To determine the protein concentration, 20 μL of enzyme was added to 200 μL of Bradford reagent, and the absorbance value was measured at OD595. Purified proteins were further analyzed using SDS-PAGE, with a protein molecular weight marker obtained from Beijing Solarbio Science & Technology Co., Ltd., Beijing, China.

### 4.5. Enzymatic Characterisation of Recombinant β-Glucosidase LQ-BG5

#### 4.5.1. Determination of β-Glucosidase Activity

The activity of β-glucosidase was assessed using cellobiose (Sangon Biotech (Shanghai) Co., Ltd., Shanghai, China) as the substrate. Briefly, 10 μL of the enzyme solution was mixed with 90 μL of an optimal pH buffer containing 1% (*w*/*v*) cellobiose (prepared in the laboratory), and the reaction proceeded for 10 min at the predetermined optimal temperature. Following this, the reaction was halted by freezing at −80 °C for 5 min. Subsequently, 10 µL of the reaction mixture was aliquoted onto a 96-well culture plate, and 200 µL of buffer from the glucose oxidase–peroxidase assay kit (BioSino Bio—Technology & Science Inc., Beijing, China) was added separately. The mixture was then incubated at 37 °C for 10 min, and the absorbance value was measured at 492 nm [45]. One unit (U) of β-glucosidase activity was defined as the amount of enzyme required to release 2 µmol of glucose per minute of hydrolysis of cellobiose.

#### 4.5.2. Optimum Temperature and Thermal Stability

To determine the optimal reaction temperature, the purified β-glucosidase was incubated for 30 min at various temperatures ranging from 20 °C to 75 °C, while maintaining optimal reaction pH conditions. The impact of the temperature on the enzyme activity was analyzed across a 5 °C gradient to ascertain the optimum reaction temperature. Thermal stability analyses commenced by subjecting the enzymes to different temperatures (50 °C, 55 °C, and 60 °C) for 10 h each, with samples collected at hourly intervals until the conclusion of the incubation period. Subsequently, the enzyme reaction was initiated, and the treated enzyme was exposed to the optimal temperature and pH conditions for 30 min to assess the residual enzyme activity of LQ-BG5. Comparing the residual activity of the enzyme allows for conclusions regarding its stability at specific temperatures and, consequently, its thermal stability. An untreated enzyme reaction mixture under standard conditions served as the control (100%).

#### 4.5.3. Optimum pH and pH Stability

The enzymatic reaction of β-glucosidase was conducted using buffers with varying pH levels to determine its optimal reaction pH. Na_2_HPO_4_-citric acid buffer at pH values ranging from 3.0 to 8.0 and Glycine-NaOH buffer at pH values of 8.0 to 10.0 were employed to react with the enzyme at the optimal reaction temperature for 30 min. The effect of the pH on the enzyme activity was analyzed to determine the optimal reaction pH. For pH stability analysis, Na_2_HPO_4_-citric acid buffer (pH 3.0–8.0) and Glycine-NaOH buffer (pH 8.0–10.0) were utilized. Initially, pure enzyme solutions were mixed with buffers of varying pH in a 1:2 ratio for each group. One group was stored at 4 °C for 12 h, another for 24 h, and the rest were left untreated. Subsequently, the residual enzyme activity of LQ-BG5 was determined by reacting the enzyme treated with pH buffers at the optimal temperature and pH for 30 min. Comparing the residual enzyme activity at each pH level can help determine its pH stability. An untreated enzyme reaction mixture under standard conditions served as the control (100%).

#### 4.5.4. Effect of Metal Ions and Chemical Reagents on Recombinant Enzymes

Metal ions and chemical reagents can either inhibit or activate enzyme activity, which is crucial in various industrial applications. In order to assess the effect of metal ions and chemical reagents on the enzyme activity, two sets of experiments were performed with the same conditions except that different concentrations of metal ions and chemical reagents were used. Metal ions (K^+^, Mg^2+^, Fe^3+^, Ca^2+^, Zn^2+^, Co^2+^, Cu^2+^, Ag^+^, Mn^2+^, Pb^2+^, and Ni^2+^) were introduced into the reaction system along with the enzyme, resulting in final concentrations of 1 mM and 10 mM, respectively. Additionally, chemical reagents including disodium ethylenediaminetetraacetic acid (EDTA), Tween-80, dithiothreitol (DTT), and sodium dodecyl sulfate (SDS) were added to achieve final concentrations of 1% and 0.1%, respectively. A reaction mixture devoid of any additives under standard conditions served as the control (100%). The residual enzyme activity following the addition of metal ions and chemical reagents was assessed at the optimal temperature and pH to analyze their effects on the enzyme activity.

#### 4.5.5. Effect of Glucose Concentration on Enzyme Activity

Using p-nitrophenyl-β-D-glucopyranoside (pNPG) as a substrate, 10 µL of LQ-BG5 was added to 125 µL of glucose solutions with varying concentrations (0 mM, 500 mM, 1000 mM, 1500 mM, 2000 mM, 2500 mM, 3000 mM), along with 10 mM pNPG (15 µL). A control group with no enzyme was included [2,45,47]. The reaction proceeded for 10 min at 55 °C and was terminated by adding 450 µL of 1 M Na_2_CO_3_. The absorbance value was measured at 405 nm [48,49]. The inhibition constant (Ki) of LQ-BG5 is determined as the concentration of glucose at which the enzyme activity is inhibited by half [47,50].

#### 4.5.6. Determination of Substrate Specificity and Kinetic Parameters

The hydrolysis product generation was assessed by introducing 1% of various substrates (cellobiose, α-lactose, pNPG, sucrose, corn cob xylan, avicel, wheat bran xylan, CMC, bagasse xylan, beech xylan) into the enzyme reaction system under optimal reaction conditions [51]. Thus, the substrate specificity of LQ-BG5 was determined. 

Kinetic parameters were determined by reacting different concentrations of cellobiose and α-lactose (0.2–2%) at the optimal temperature and pH for 5 min and 10 min, respectively. The Michaelis–Menten equation was employed to calculate the kinetic parameters (Vmax and Km) of LQ-BG5, and a Lineweaver–Burk double-inverse plot was constructed [44].

### 4.6. Statistical Analysis

Unless otherwise stated, all assays were triplicated, and the mean was used in all analyses. The results were analyzed by SPSS 20.0 and expressed as means ± SEM. Statistical analyses were performed by using one-way ANOVA followed by Tukey’s test for the comparison of multiple treatment groups. In all comparisons, *p* values < 0.05 were considered statistically significant [52].

## 5. Conclusions

In summary, a β-glucosidase functional gene belonging to the GH1 family, designated as *lq-bg5*, was isolated from the Hehua hot spring in Tengchong City, Yunnan Province, China, through metagenomic methods. This gene was successfully cloned and expressed in *E. coli* DH5α. The recombinant enzyme (LQ-BG5) was purified and characterized. Its optimal temperature and pH were 55 °C and 4.6, respectively. The enzyme characteristics indicate that LQ-BG5 thrives at high temperatures (45–65 °C) and in acidic conditions (pH 4.6–6.6), maintaining stability across a range of temperatures (50–60 °C) and pH levels (5.0–10.0). The enzyme is also resilient to heavy metals such as Co^2+^, Cu^2+^, and Ni^2+^, and to typical inhibitors including EDTA and glucose. Remarkably, LQ-BG5 possesses both β-glucosidase and β-galactosidase activities. Its thermal stability and resistance to glucose are on par with other β-glucosidases from the metagenomic DNA of hot springs. This suggests that native environments of enzymes may determine their enzymatic properties, while extreme environments are more likely to produce enzymes with specific traits during evolution. Given its origin from a hot spring and its robust characteristics, LQ-BG5 is particularly well suited for industrial applications that require resilience to high temperatures, acidity, and glucose, making it a promising candidate for lignocellulosic biomass conversion and for use in the food and feed sectors.

## Figures and Tables

**Table 1:** Effects of metal ions and chemical reagents on the activity of recombinase LQ-BG5.

	Relative Activity	
Effectors	Effector Concentration (mM)	
	1 mM	10 mM
Control	100.0 ± 0.6	100.0 ± 0.6
K^+^	101.2 ± 1.9	99.1 ± 3.5
Mg^2+^	89.6 ± 1.7 **	88.4 ± 0.2 **
Fe^3+^	88.9 ± 3.3 **	9.4 ± 1.4 **
Ca^2+^	100.9 ± 2.0	94.3 ± 4.2 *
Zn^2+^	92.1 ± 2.1 **	92.2 ± 0.7 **
Co^2+^	99.3 ± 2.5	0 **
Cu^2+^	106.4 ± 2.1 **	98.9 ± 1.0
Ag^+^	88.5 ± 1.1 **	0 **
Mn^2+^	85.1 ± 0.6 **	21.5 ± 1.8 **
Pb^2+^	86.9 ± 4.7 **	67.1 ± 4.4**
Ni^2+^	107.0 ± 1.8 **	86.8 ± 2.4 **
	Effector concentration (W/V)	
	0.1%	1%
EDTA	103.8 ± 3.2	95.8 ± 4.0
Tween-80	86.0 ± 0.9 **	84.4 ± 1.5 **
SDS	0.6 ± 0.1 **	0 **
DTT	90.6 ± 5.7 **	87.0 ± 1.6 **

Control, no reagents were added to the reaction. Values represent the mean of three biological replicates (mean ± SEM). ** *p* < 0.01, * *p* < 0.05. The primary activity was taken as 100%. In total, 100% = 7.0 ± 2.1 U/mg.

**Table 2:** Substrate specificities of LQ-BG5.

Substrate	Special Activity (U/mg)
cellobiose	7.0 ± 2.1
α-lactose	13.7 ± 1.5
pNPG	24.1 ± 10.8
sucrose	0
corn cob xylan	0
avicel	0
wheat bran xylan	0
CMC	0
bagasse xylan	0
beechwood xylan	0

**Table 3:** Comparison of some enzymatic properties of β-glucosidase.

Source of Enzyme	Optimum pH	Optimum Temperature °C	Thermostability	Km (mM) Cellobiose	Glucose Tolerance	References
LQ-BG5	4.6	55	89% residual activity after 10 h incubation at 50 °C	3.11	69% relative activity at 2 M	This study
*Thermoanaerobacterium* *thermosaccharolyticum* DSM 571	6.4	70	80% residual activity after 2 h incubation at 60 °C	7.9	30% relative activity at 1 M	[24]
Shenzhen Mangrove Reserve metagenomic	6	40	20% residual activity after 30 min incubation at 45 °C	ND	70% relative activity at 3.6 M	[40]
*Trichoderma harzianum*	6	40	CD thermal-induced unfolding, Tm = 49 °C	1.22	30% relative activity at 0.8 M	[41]
*B. subtilis* RA10	5	50	68.32% residual activity after 48 h incubation at 50 °C	ND	70% relative activity at 1 M	[1]
*Meyerozyma guilliermondii*	3.5–5.5	55	70% residual activity after 80 min of incubation at 55 °C	ND	40% relative activity at 1 M	[29]
*Thermococcus* sp.	5.5–6.5	78	50% residual activity after 55 min incubation at 78 °C	16.48	100% relative activity at 4 M	[38]
*Jeotgalibacillus malaysiensis*	7	65	50% residual activity after 35 min incubation at 65 °C	ND	80% relative activity at 2.5 M	[42]
*Leifsonia* sp. ZF2019 (Bgl1973)	7	50	80% residual activity after 1 h incubation at 40 °C	ND	83% relative activity at 1 M	[31]
Hot spring metagenomic	5	70	50% residual activity after 32 h incubation at 60 °C	16.55	40% relative activity at 5 M	[23]

## Data Availability

Original contributions presented in this study are included in the article/Supplementary Materials. The nucleotide sequence of the LQ-BG5 gene was submitted to GenBank (https://www.ncbi.nlm.nih.gov/nuccore/OR724869) (accessed on 23 October 2023). Further inquiries can be directed to the corresponding authors.

## Supplementary Materials

The following supporting information can be downloaded at: https://www.mdpi.com/article/10.3390/molecules29051017/s1, Figure S1: PCR results of β-glucosidase gene (*lq-bg5*); Figure S2: Screening of *E. coli* clones containing β-glucosidase gene (*lq-bg5*) under UV light; Figure S3: Three-dimensional model of β-glucosidases LQ-BG5; Figure S4: Complete SDS-PAGE.
