# Supplementary material for: A Recombinant Thermophilic and Glucose-Tolerant GH1 β-Glucosidase Derived from Hehua Hot Spring
Source: Molecules. 2024 Feb 26;29(5):1017. doi: 10.3390/molecules29051017 (PMC10934247; doi:10.3390/molecules29051017)
Supplement: Supplementary file 1 [file molecules-29-01017-s001.zip › molecules-2829515-supplementary.pdf]

Supplementary material

List of the supporting materials:

Figures: Supplementary Figure S1, Supplementary Figure S2, Supplementary Figure S3 and Supplementary Figure S4.

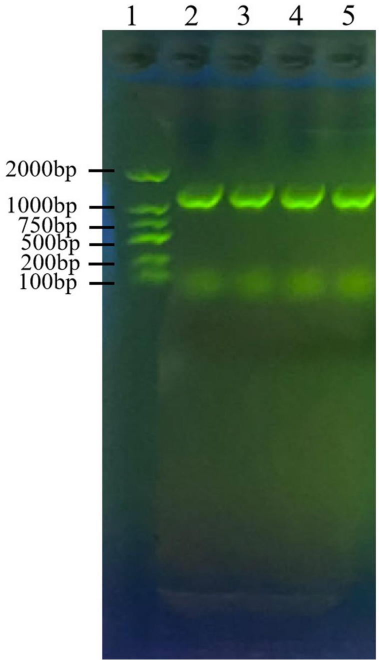

Supplementary Figure S1. PCR results of β-glucosidase gene (*lq-bg5*). Lane 1, DNA marker, mass indicated on the left; lane 2-5, DNA fragment of *lq-bg5* obtained by PCR amplification.

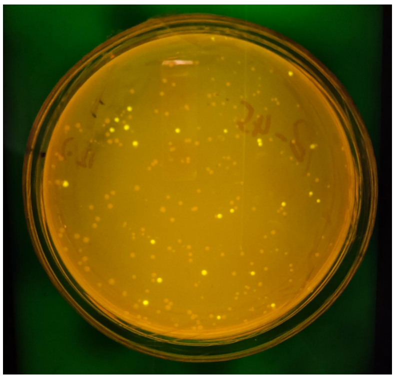

Supplementary Figure S2. Screening of *E. coli* clones containing β-glucosidase gene (*lq-bg5*) under UV light. The negative clones have green fluorescence, while the positive clones have no green fluorescence.

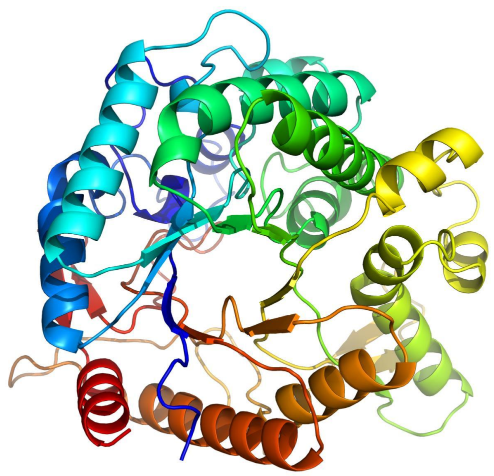

Supplementary Figure S3. Three-dimensional model of β-glucosidases LQ-BG5.

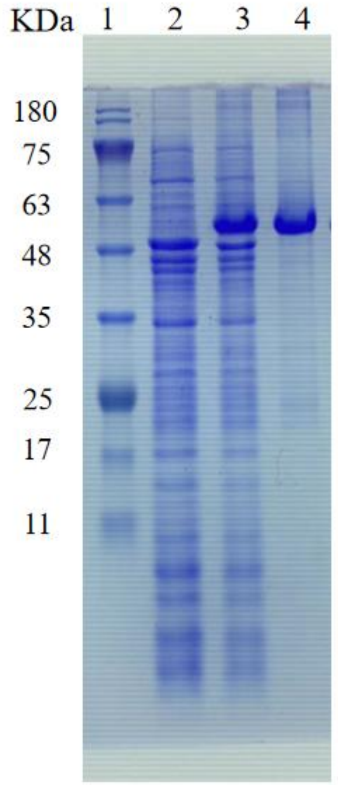

Supplementary Figure S4. Complete SDS-PAGE. Lane 1, protein molecular weight marker; Lane 2, protein of *E. coli* DH5α prior to induction; Lane 3, total protein of *E. coli* DH5α/pSHY211- LQ-BG5; Lane 4, purified LQ-BG5 protein.
